# Surface Cracking Mechanism of SP2215/Stellite-6 Components Fabricated by Laser-Directed Energy Deposition During High-Temperature Service

**DOI:** 10.3390/ma19183911

**Published:** 2026-09-15

**Authors:** Pengcheng Che, Nan Wang, Wei Wu, Qiushi Li, Liwen Rao, Li Yang, Jian Dong, Zhiliang Ning, Chenglei Fan, Yongjiang Huang

**Affiliations:** 1 School of Materials Science and Engineering, Harbin Institute of Technology, Harbin 150006, China; 2 Harbin Energy Innovation Digital Technology Co., Ltd., Harbin 150028, China; 3 Harbin No. 69 Middle School, Harbin 150001, China; 4 State Key Laboratory of Low-Carbon Thermal Power Generation Technology and Equipment, Harbin Boiler Company Limited, Harbin 150046, China; raolw@hbc.com.cn

**Keywords:** laser-directed energy deposition, Stellite-6 coating cracking, M23C6 carbide, elemental diffusion, surface hardness

## Abstract

Laser-directed energy deposition (L-DED) is a key technology for fabricating wear-resistant Stellite-6 coatings on power-plant components. This study aims to clarify the surface cracking mechanism of L-DED Stellite-6 coatings deposited on 22Cr15Ni3.5CuNbN (SP2215) boiler tubes during long-term high-temperature service. The coatings were exposed at 650 °C for up to 5000 h, and their microstructural evolution and mechanical degradation were systematically characterized using SEM/EDS, TEM, EBSD, microhardness testing, and impact testing. High-temperature service induces the decomposition of M_23_C_6_ precipitates at dendrite boundaries, releasing Cr, C, and W atoms that subsequently migrate toward the coating surface. Owing to the rapid interstitial diffusion of C, a carbon-enriched surface region preferentially develops, accompanied by progressively increasing coverage of surface Cr_2_O_3_ and subsurface M_23_C_6_. Consequently, the surface hardness increases from 441.6 HV0.1 at 0 h to 613.1 HV0.1 after 5000 h, whereas the impact-absorbed energy decreases from 82.8 ± 4.2 J to 5.9 ± 2.5 J. An increase of 1 HV0.1 in hardness corresponds to an approximately 0.448 J reduction in impact-absorbed energy. Mechanistically, local stress concentration associated with Cr_2_O_3_ formation, interfacial sliding promoted by lattice mismatch, and crack nuclei originating from pores between chain-like M_23_C_6_ precipitates collectively promote crack initiation and propagation. These results demonstrate that precipitate decomposition, elemental redistribution, and subsequent oxide/carbide evolution govern the progressive surface embrittlement and cracking of L-DED Stellite-6 coatings during long-term high-temperature service. This study provides mechanistic insight into the coupling between microstructural evolution and surface failure and offers a theoretical basis for microstructural regulation and long-term reliability assessment of wear-resistant Co-based coatings used in power-plant components.

## 1. Introduction

L-DED-fabricated Stellite-6 coatings have been widely used for surface strengthening of critical metallic components in power plants, such as boiler tube outer walls, valve sealing surfaces, and hydraulic turbine runners, owing to their excellent wear resistance, corrosion resistance, and high-temperature stability. Taking boiler tubes as an example, L-DED Stellite-6 coatings can effectively mitigate tube-wall wear failure caused by pulverized coal, high-temperature flue gas, and solid-particle erosion. Compared with conventional surface-strengthening techniques such as flame spraying and electroplating, L-DED enables metallurgical bonding between the coating and the substrate, thereby improving the coating adhesion strength and service life [1,2]. However, during long-term high-temperature service at 650 °C, Stellite-6 coatings still suffer from reduced microstructural stability and surface cracking failure [3,4]. In boiler-tube service environments in particular, the coating is continuously subjected to erosion and impact from pulverized-coal flow and high-temperature flue gas, as well as cyclic thermal stresses induced by the flow of high-pressure steam inside the tube, making surface cracking prone to occur. Cracks may further propagate through the tube wall, triggering cascading failures such as steam leakage. Therefore, elucidating the microstructural evolution and cracking failure mechanism of L-DED Stellite-6 coatings during high-temperature service is of great engineering significance.

Stellite-6 is a typical Co-based wear-resistant alloy, and its excellent wear resistance is closely associated with the crystallographic transformation of the Co matrix, low stacking-fault energy, and carbide strengthening. Under stress or temperature, the Co matrix can transform from the face-centered cubic (FCC) structure to the hexagonal close-packed (HCP) structure, which is generally beneficial for improving wear resistance [5,6,7]. In addition, the carbide content, morphology, and distribution in the alloy markedly affect the strength, hardness, and wear resistance of the coating [8,9,10]. Previous studies have shown that high-temperature aging alters the phase constitution and mechanical properties of Stellite-6 alloys. Avneesh Kumar et al. [11] found that thermal aging at 600 °C increased the HCP-Co fraction in Stellite-6 coatings and raised the microhardness from 635 ± 75 HV_0.3_ to 740 ± 50 HV_0.3_. However, HCP-Co has poor ductility and may therefore reduce coating toughness [12,13,14]. Aprameya et al. [15] further reported that repeated fatigue loading during high-temperature service can induce cracking and spallation of Stellite-6 coatings. These findings indicate that increased coating hardness under high-temperature service does not necessarily imply improved service reliability; instead, microstructural hardening may be intrinsically linked to embrittlement-induced cracking.

For carbide-strengthened Co-based superalloys, MC, M_23_C_6_, and M_6_C are the dominant carbide types. In cast Stellite alloys, M_23_C_6_ usually precipitates at grain boundaries or interdendritic regions during slow cooling [16,17,18]. Fine and dispersed M_23_C_6_ carbides can, to some extent, inhibit grain-boundary sliding and thereby improve creep-rupture strength. However, when M_23_C_6_ becomes coarsened, continuous, or chain-like, it may weaken grain-boundary cohesion and provide preferential paths for crack initiation and propagation [19,20,21]. Therefore, the precipitation, coarsening, decomposition, and redistribution of M_23_C_6_ during high-temperature service are key factors governing surface cracking in Stellite-6 coatings.

In our previous work, we systematically investigated the microstructural evolution and thermo-mechanical coupling characteristics of the interfacial region in L-DED SP2215/Stellite-6 components during service. Although M_23_C_6_ in the surface and interfacial regions exhibits a similar coarsening mechanism during service, both being associated with the migration and reprecipitation of Cr and C atoms released by the high-temperature decomposition of unstable precipitates along the Stellite-6 dendritic boundaries, the two regions differ significantly in terms of elemental-migration boundary conditions and M_23_C_6_ enrichment behavior, oxidation environment and crack-initiation mechanism, as well as mechanical-property response and ultimate failure mode.

In addition to carbides, the formation and evolution of surface Cr_2_O_3_ oxides may also affect coating cracking. Previous studies have shown that grain-boundary sliding in thermally grown Cr_2_O_3_ scales can be activated as an accompanying mechanism of diffusional creep and may induce scale delamination [22,23]. Meanwhile, the local strain rate at grain boundaries in Cr_2_O_3_ scales is markedly higher than that within grains, and the grain-boundary regions exhibit lower toughness, making crack nucleation more likely at oxide particle interfaces [24]. In addition, Cr_2_O_3_ ceramics have relatively low fracture toughness, with *K_IC_* of approximately 4.0–4.6 MPa·m^1/2^, indicating their susceptibility to interfacial cracking and crack propagation under high-temperature stress [25,26]. Therefore, during high-temperature service, the Cr_2_O_3_ phase may become a preferential site for surface crack initiation due to grain-boundary sliding, reduced interfacial bonding, and local stress concentration.

In summary, although previous studies under long-term service conditions at 650 °C have revealed the microstructural evolution of L-DED Stellite-6 during short-term thermal service, as well as the relationship between M_23_C_6_ carbide evolution and cracking behavior, the maximum service duration investigated has generally not exceeded 1000 h. This greatly limits the understanding of microstructural and property evolution during more prolonged service, especially considering that a service duration of at least 5000 h is commonly regarded as an important time scale for evaluating the long-term service performance of power-plant components. Meanwhile, the long-term evolution of Cr_2_O_3_ oxides on the surface of L-DED Stellite-6 and their relationship with cracking behavior have not yet been systematically investigated, although Cr_2_O_3_ oxides also have a significant influence on the formation of surface microcracks. In addition, the relationship between the evolution mechanisms of surface M_23_C_6_ and Cr_2_O_3_ phases and the thermo-mechanical response of the surface region, as well as their synergistic effects on surface hardening, impact-toughness degradation, and cracking failure, remain unclear and require further systematic investigation.

Accordingly, this study focuses on L-DED Stellite-6 coatings deposited on SP2215 boiler tubes and investigates their surface microstructural evolution, phase-constitution changes, and mechanical-property degradation during long-term service at 650 °C up to 5000 h. Particular emphasis is placed on the roles of M_23_C_6_ carbides and Cr_2_O_3_ oxides in surface cracking of the coating. The results provide a theoretical basis for failure analysis, life assessment, and crack-resistant design of L-DED Stellite-6 wear-resistant coatings.

## 2. Experimental Details

### 2.1. Materials and Processing

**Figure 1:**
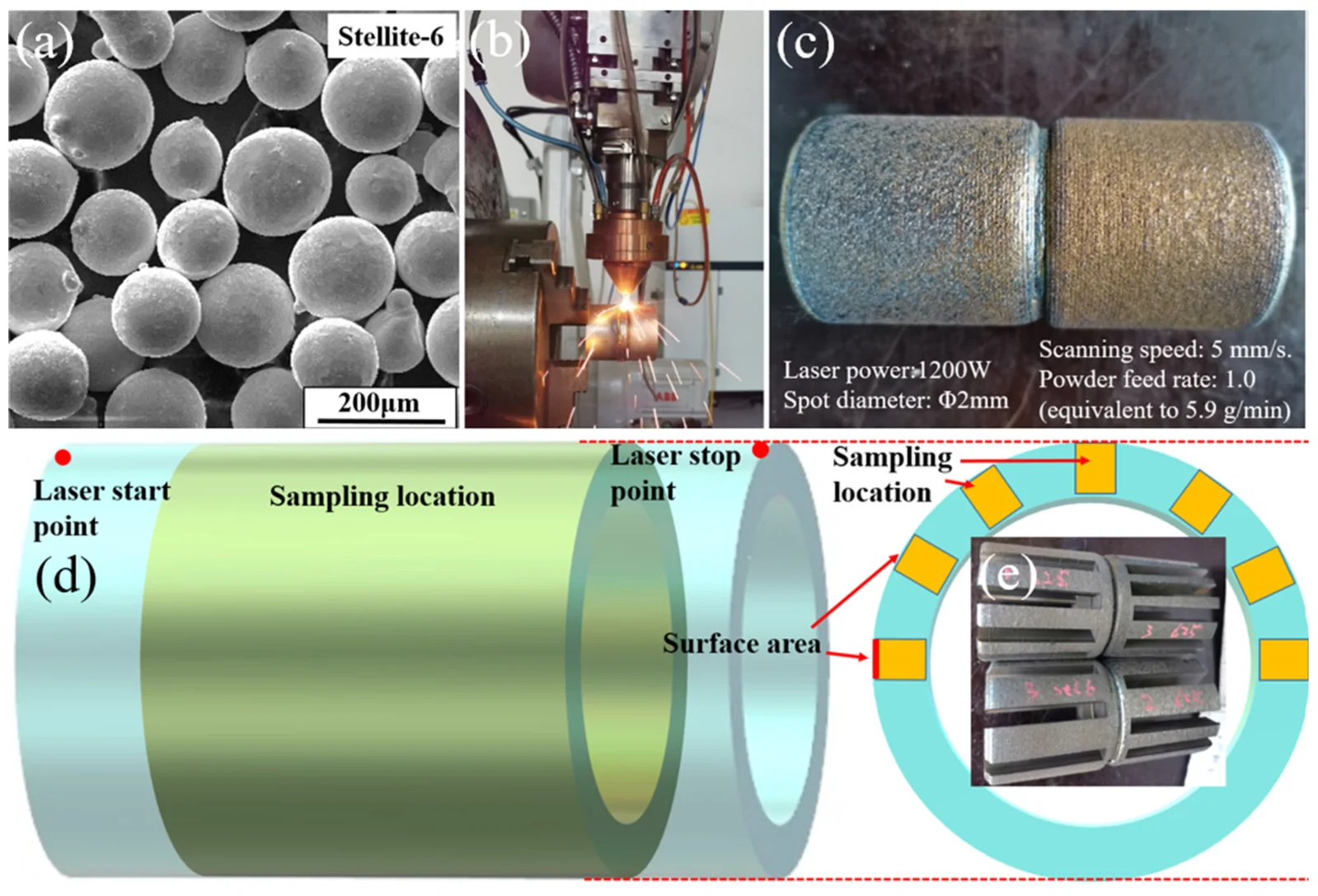
Powder morphology, macroscopic morphology, and chemical compositions of the L-DED SP2215/Stellite-6 component: (**a**) Stellite-6 alloy powder; (**b**) L-DED fabrication of the SP2215/Stellite-6 component; (**c**) macroscopic morphology and chemical composition table of the component; (**d**) sampling location; (**e**) macroscopic morphology of the sampling location.

The Stellite-6 powder used for L-DED was supplied by Höganäs, Höganäs, Sweden, and produced by gas atomization, with a particle size of 53–150 μm. The substrate was an SP2215 austenitic stainless-steel tube with dimensions of φ43 × 6 mm. Figure 1 shows the powder morphology used for L-DED, the morphology of the fabricated specimens, and the corresponding specimen sampling locations. Figure 1a shows the morphology of the Stellite-6 alloy powder. Figure 1b illustrates the L-DED fabrication process of the SP2215/Stellite-6 component. The specimen was fabricated using a laser power of 1200 W, a spot diameter of 2 mm, a powder feed rate of 5.9 g/min, and a scanning speed of 5 mm/s, producing a coating with a thickness of 4 mm. Figure 1c shows the morphology of the fabricated SP2215/Stellite-6 component with dimensions of φ51 × 10 mm. Figure 1d shows the schematic illustration of the sampling locations on the L-DED tubular specimen, and Figure 1e presents the macroscopic morphology of the sampled specimens. Table 1 lists the main chemical compositions of Stellite-6 and SP2215.

The specimens used in this study were block samples sectioned from the as-fabricated L-DED tubular components, with the sampling locations shown in Figure 1. To ensure representativeness and avoid local microstructural and compositional variations caused by transient heat-input fluctuations during laser start and stop, all SEM/EDS characterizations and subsequent analyses were conducted in the middle region of the tubular specimens. This region corresponds to the stable deposition stage and therefore reliably represents the microstructure and elemental distribution under steady-state L-DED conditions.

### 2.2. High Temperature Service

The L-DED-fabricated SP2215/Stellite-6 specimens were placed in a high-temperature-resistance furnace at 650 °C, corresponding to the service temperature of boiler tube panels. High-temperature air was used as the furnace atmosphere to simulate the boiler environment after pulverized-coal combustion. Seven long-term service durations were selected: (a) 0 h, (b) 100 h, (c) 300 h, (d) 500 h, (e)1000 h, (f) 3000 h, and (g) 5000 h.

### 2.3. Sample Preparation and Characterization

SEM specimens were obtained from the L-DED samples by wire electrical discharge machining (WEDM), followed by mechanical polishing to a mirror-like surface. The polished specimens were etched for 10 s using a solution composed of 4 mL distilled water, 3 mL ethanol, and 1 mL HNO_3_. Microstructural characterization was carried out using a field-emission scanning electron microscope (Apollo 300, CamScan, Cambridge, UK) equipped with an energy-dispersive X-ray spectroscopy (EDS) system.

For TEM examination (JEOL JEM-2100, JEOL Ltd., Tokyo, Japan), 0.3 mm thick slices were first cut by WEDM and mechanically thinned to a thickness below 100 μm. Subsequently, 3 mm diameter discs were punched with a precision disc puncher (Gatan Model 656, Gatan, Inc., Pleasanton, CA, USA) and further thinned by ion milling to below 150 nm to obtain electron-transparent specimens.

Unnotched Charpy impact specimens measuring 10 mm × 10 mm × 55 mm were machined from the L-DED-fabricated SP2215/Stellite-6 samples by WEDM in accordance with GB/T 229–2020 [27]. Impact tests were performed using an NI500 digital pendulum impact testing machine.

Specimens for hardness measurements were also sectioned from the L-DED-fabricated SP2215/Stellite-6 samples by WEDM according to GB/T 4340.1–2024 and ISO 14577 [28,29]. Microhardness testing was conducted using a Vickers microhardness tester under a load of 0.9807 N, corresponding to HV_0.1_.

## 3. Results

### 3.1. Subsurface Microstructural Evolution and M_23_c_6_ Formation Mechanism

**Figure 2:**
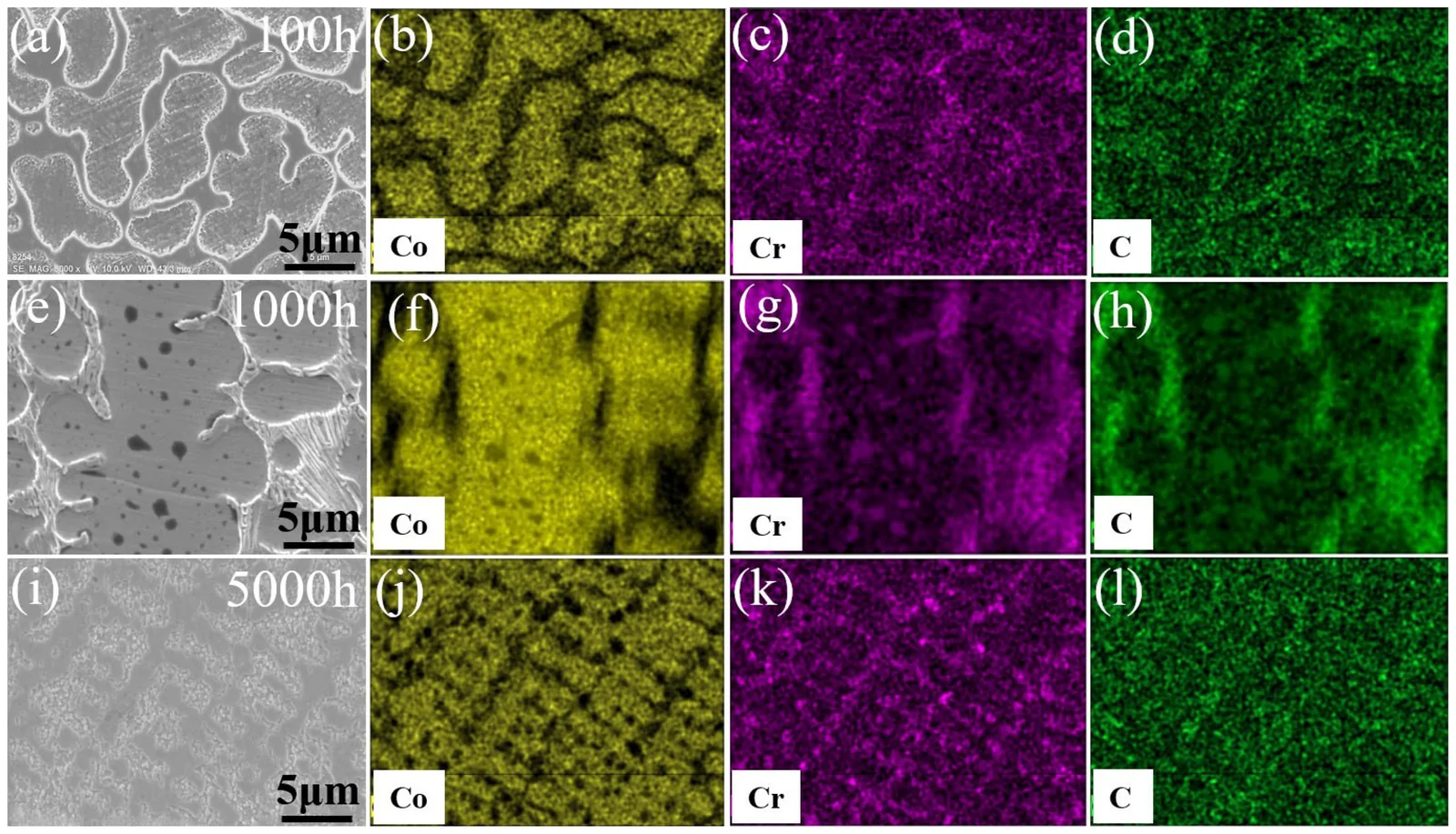
SEM and EDS analysis results of L-DED Stellite-6 after service for 100, 1000, and 5000 h: (**a**–**d**) 100 h; (**e**–**h**) 1000 h; (**i**–**l**) 5000 h.

Figure 2 shows SEM images and EDS maps of the microstructural evolution at the dendrite boundaries of Stellite-6 during service. Figure 2a shows the SEM morphology of the L-DED Stellite-6 dendrite boundaries after 100 h of service, and Figure 2b–d presents the corresponding EDS elemental maps. The dendrite boundaries are mainly covered by Cr- and C-rich M_23_C_6_ phases, with an average precipitate-band width of approximately 1.3 μm, whereas the Cr and C contents within the dendrite cores are relatively low.

Figure 2e shows the SEM morphology of the L-DED Stellite-6 dendrite boundaries after 1000 h of service, and Figure 2f–h presents the corresponding EDS elemental maps. At this stage, the boundary precipitates have undergone pronounced decomposition, exposing the original fine dendrites. In local regions, Cr and C released from the decomposed dendrite-boundary precipitates recombine at heterogeneous nucleation sites within the dendrite cores to form M_23_C_6_, with an average size of approximately 1.1 μm.

Figure 2i shows the SEM morphology of the L-DED Stellite-6 dendrite boundaries after 5000 h of service, and Figure 2j–l presents the corresponding EDS elemental maps. At this stage, the boundary precipitates are markedly decomposed, refined, and transformed into chain-like features, with the average precipitate-band width decreasing to approximately 0.7 μm. Cr and C are uniformly dispersed throughout the microstructure.

These results indicate that, as the thermal service duration increases from 100 to 5000 h, C and Cr progressively diffuse from the precipitate bands at the dendrite boundaries into the dendrite interiors, resulting in refinement of the boundary precipitate bands.

**Figure 3:**
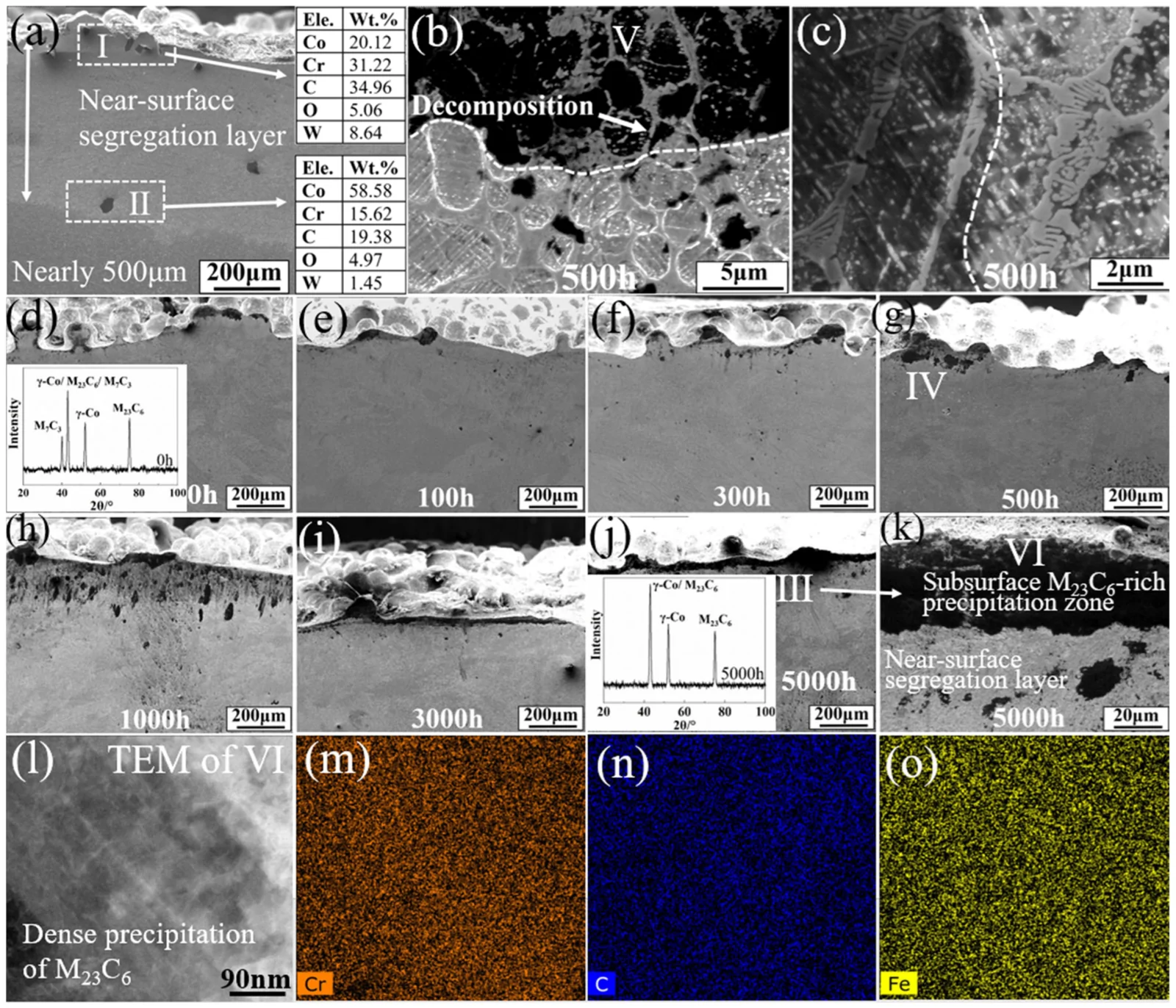
Subsurface morphological evolution of L-DED Stellite-6 during service: (**a**) Subsurface morphology and EDS analysis after 0 h of service; (**b**) interfacial morphology of the M_23_C_6_-rich region after 500 h of service; (**c**) decomposition morphology of dendrite-boundary precipitates; (**d**–**k**) subsurface morphological evolution during 0–5000 h of service; (**l**) TEM image of region VI; (**m**–**o**) EDS maps of (**l**).

Figure 3 shows the morphological evolution of the subsurface region of L-DED Stellite-6 during service. Figure 3a presents the near-surface morphology and EDS analysis of the as-deposited L-DED Stellite-6 coating. SEM observations reveal a continuous layered region with an average thickness of approximately 500 μm beneath the surface. EDS results indicate that this region is a near-surface segregation layer enriched in Cr, C, W, and O. In the as-deposited state, a large number of blocky M_23_C_6_ precipitates are already present in this region, whereas they are not observed in the non-segregated region away from the near surface. Within this layer, the contents of Cr, C, W, and O increase toward the surface, indicating that the degree of elemental segregation becomes more pronounced with decreasing distance from the surface.

Figure 3d–k show the subsurface morphologies of L-DED Stellite-6 after thermal service from 0 to 5000 h. Figure 3d shows that, at 0 h, M_23_C_6_ particles are present only in local subsurface regions, and the average thickness of the M_23_C_6_ precipitation zone is only 3.6 μm. Figure 3e–j show that the coverage of subsurface precipitates gradually increases with service time and progressively forms a black-contrast precipitation band, namely the subsurface M_23_C_6_-rich precipitation zone. After 3000 h of service, the subsurface region is fully covered by the M_23_C_6_ precipitation zone, with a thickness of approximately 42 μm. After 5000 h, the thickness of the subsurface M_23_C_6_ precipitation zone reaches approximately 48 μm. Meanwhile, Figure 3d,j present the XRD results of the surface region after 0 h and 5000 h of service, respectively. The XRD analysis shows that, with increasing high-temperature service time, the predominant precipitate phases in the coating gradually evolve from M_23_C_6_/M_7_C_3_ in the as-deposited condition at 0 h to M_23_C_6_ after 5000 h of service. Notably, M_23_C_6_ persists throughout the entire high-temperature service period and remains the dominant precipitate phase.

Figure 3b shows a magnified view of region IV in Figure 3g, and Figure 3c shows a higher-magnification view of region V in Figure 3b. A distinct boundary is observed between the M_23_C_6_ precipitation zone and the non-M_23_C_6_ precipitation zone in the subsurface region after 500 h of service. In the precipitation zone, the precipitates at the Stellite-6 dendrite boundaries have undergone pronounced decomposition, with lamellar M_23_C_6_ remaining along the boundaries, whereas the boundary precipitates outside the surface-affected region show almost no decomposition. At this stage, the average width of the undecomposed boundary precipitate band is 1.92 μm, whereas that of the decomposed band is approximately 0.86 μm. Combined with the analysis in Figure 2, this value is close to the dendrite-boundary width in the Stellite-6 layer after 5000 h of service.

Figure 3l shows the TEM results of the subsurface M_23_C_6_ precipitation zone in region VI. The microstructure is densely covered with M_23_C_6_ phases, with continuous voids between adjacent phases. The results in Figure 3m–o show that this region is highly enriched in Cr, C, and Fe, which are typical constituents of the M_23_C_6_ phase.

**Figure 4:**
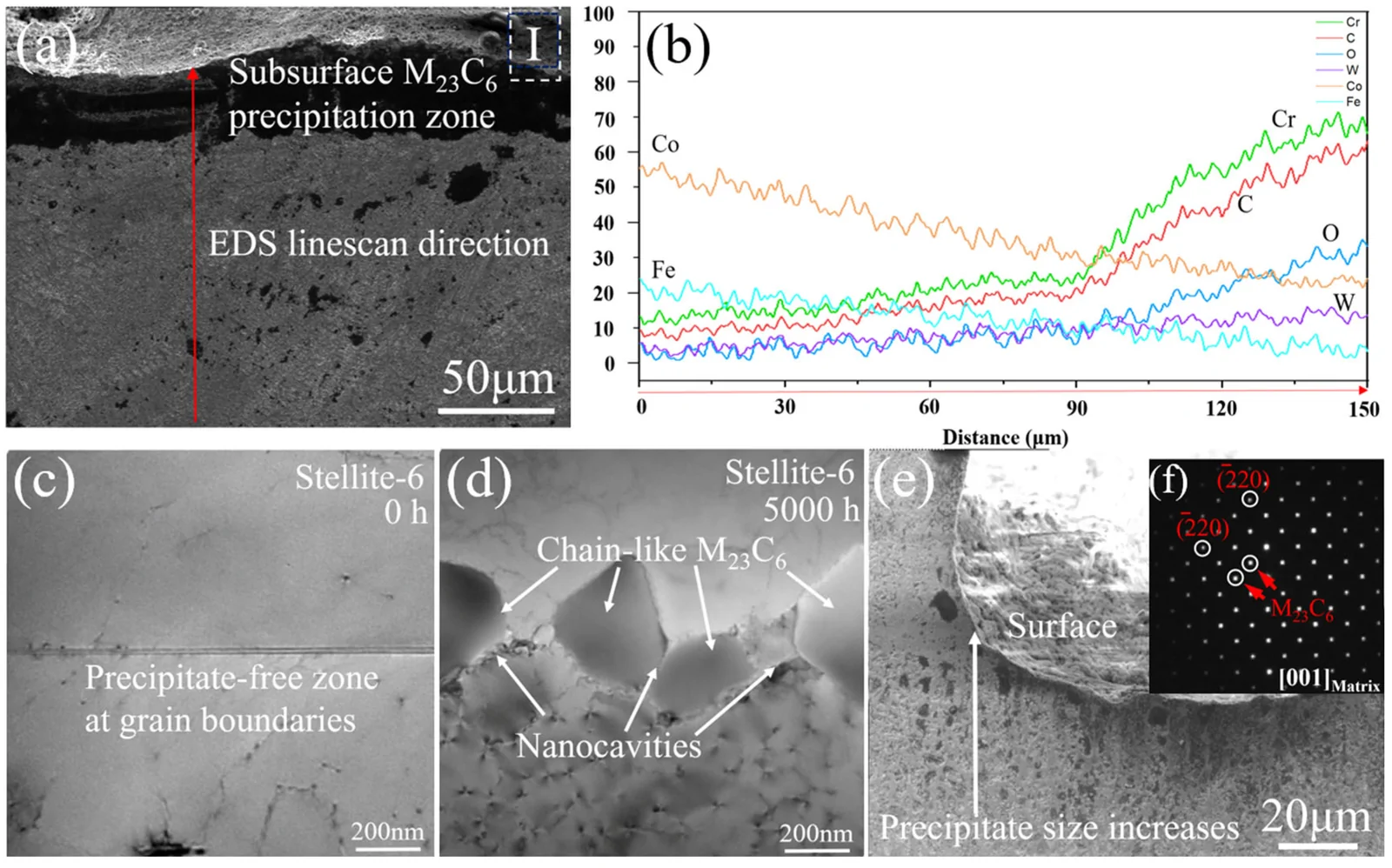
SEM, EDS and TEM analysis of the surface of L-DED SP2215/Stellite-6 during service. (**a**) Morphology of the surface M_23_C_6_ precipitation band; (**b**) EDS line-scan analysis results; (**c**) TEM of 0 h; (**d**) TEM of 5000 h; (**e**) locally magnified morphology of the surface region; (**f**) diffraction pattern of M_23_C_6_ phase.

Figure 4a,b,e provide further SEM characterization and EDS line-scan analyses of the surface region shown in Figure 3k. The results indicate that the precipitate size gradually increases as the analyzed position approaches the surface. The EDS line-scan profiles show progressively increasing intensities of Cr, C, O, and W, whereas the signals of Co and Fe gradually decrease. The signals of Cr and C begin to increase markedly at approximately 60 μm from the surface. This compositional evolution is consistent with the proposed migration of Cr and C toward defect-enriched regions near the surface during high-temperature service. Notably, the O signal begins to increase markedly within approximately 50 μm of the surface, indicating that the near-surface region undergoes more pronounced oxidation upon exposure to high-temperature air. The higher oxygen content further promotes the formation and enrichment of Cr_2_O_3_.

Figure 4c,d,f present the TEM characterization results of the surface regions corresponding to Figure 3d,j after 0 h and 5000 h of service, respectively. At 0 h, the surface grain boundaries remain nearly closed, with only a few local nucleation sites of precipitates being observed and no pronounced precipitation or agglomeration. Accordingly, both the grain-boundary precipitate coverage and the interparticle-gap coverage are approximately zero. After 5000 h of service, the grain boundaries are extensively covered by large chain-like M_23_C_6_ precipitates. Owing to the relatively high defect density at the dendritic boundaries, which provides preferential pathways for elemental diffusion and precipitation, the coarse M_23_C_6_ precipitates are predominantly distributed along the dendritic boundaries. Their average size is approximately 200–300 nm, while the interparticle gaps can reach approximately 50–200 nm. Quantitative analysis shows that the M_23_C_6_ precipitate coverage along the grain boundaries reaches approximately 70%, whereas the interparticle-gap coverage is approximately 10%. With prolonged high-temperature service, the increasing pores and gaps between adjacent precipitates provide favorable propagation paths for surface microcracks, promoting their evolution into Mode I opening cracks and thereby significantly increasing the susceptibility of the component to cracking.

**Figure 5:**
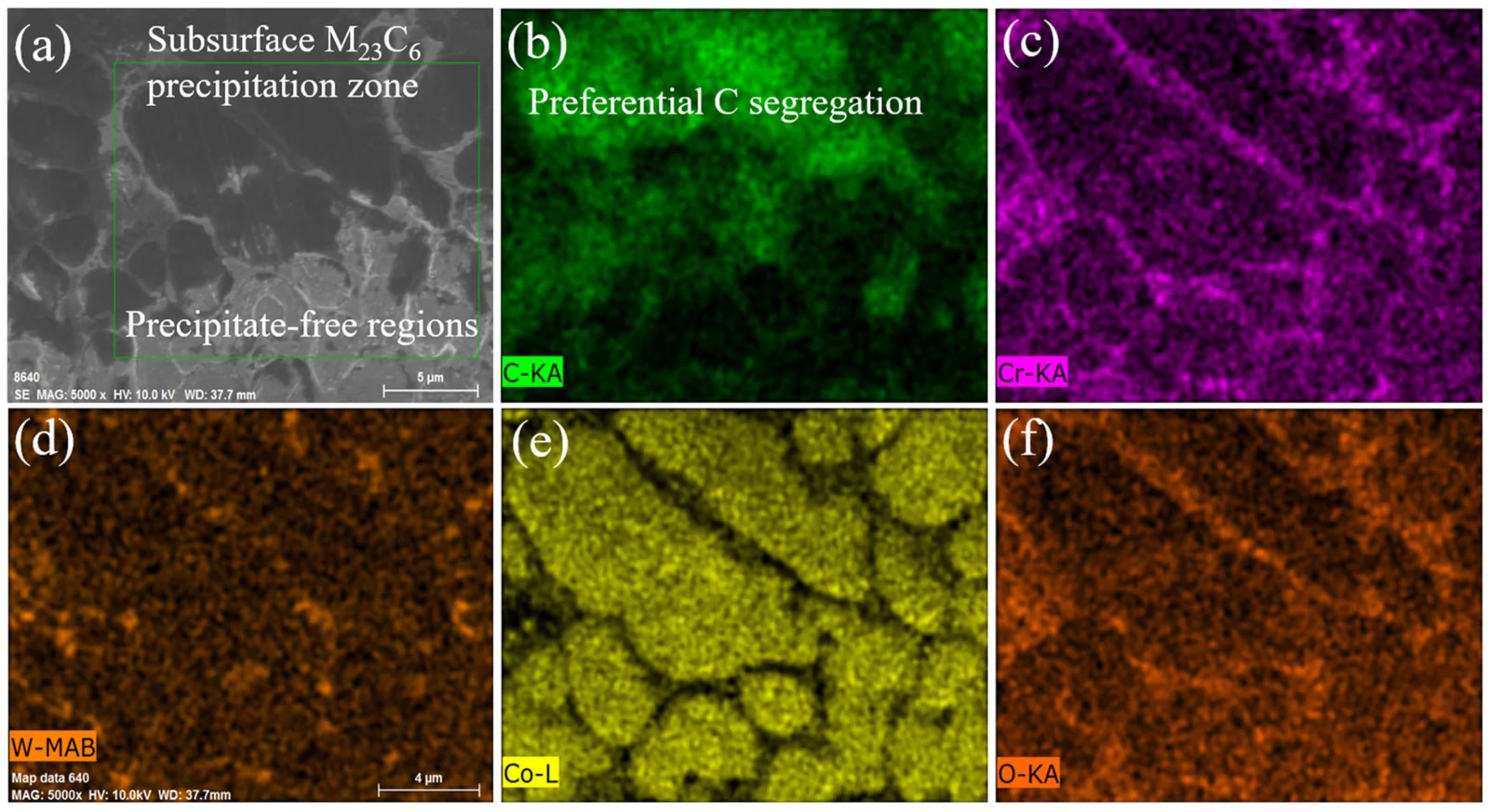
Morphology and elemental distribution of the surface M_23_C_6_ precipitation band after 500 h of service: (**a**) SEM of subsurface; (**b**) C; (**c**) Cr; (**d**) W; (**e**) Co; (**f**) O.

To further elucidate the formation mechanism of surface cracks from the perspective of compositional evolution in the surface region during high-temperature service, SEM and EDS analyses in Figure 5 were performed on the subsurface M_23_C_6_ precipitation band after 500 h of service. The 500 h condition was selected for detailed analysis because, at this stage, the precipitates along the dendritic boundaries within the Stellite-6 deposited layer had begun to undergo pronounced decomposition, whereas the subsurface region had not yet been completely covered by M_23_C_6_ formed through the migration and reprecipitation of Cr and C. This condition therefore allows for the microstructural and compositional differences between the precipitated and insufficiently precipitated regions to be distinguished more clearly.

Furthermore, EDS elemental mapping was performed across the boundary between the precipitated and insufficiently precipitated regions in the subsurface zone. The results show that the dark-contrast region near the surface exhibits pronounced C enrichment, extending across the dendritic boundaries and into the grain interiors. In contrast, no obvious C enrichment is observed within the grain interiors of the non-dark-contrast region, where Cr and C remain predominantly concentrated near the dendritic boundaries. These results indicate that, during the early stage of precipitate decomposition within the Stellite-6 layer, interstitial C atoms, owing to their relatively high diffusivity, preferentially migrate toward and accumulate in the subsurface region. This C enrichment subsequently promotes the migration of Cr and W toward the same region, thereby providing favorable conditions for the subsequent formation and growth of Cr- and C-rich precipitates such as M_23_C_6_.

### 3.2. Surface Microstructural Evolution and Cr_2_o_3_ Formation Mechanism

**Figure 6:**
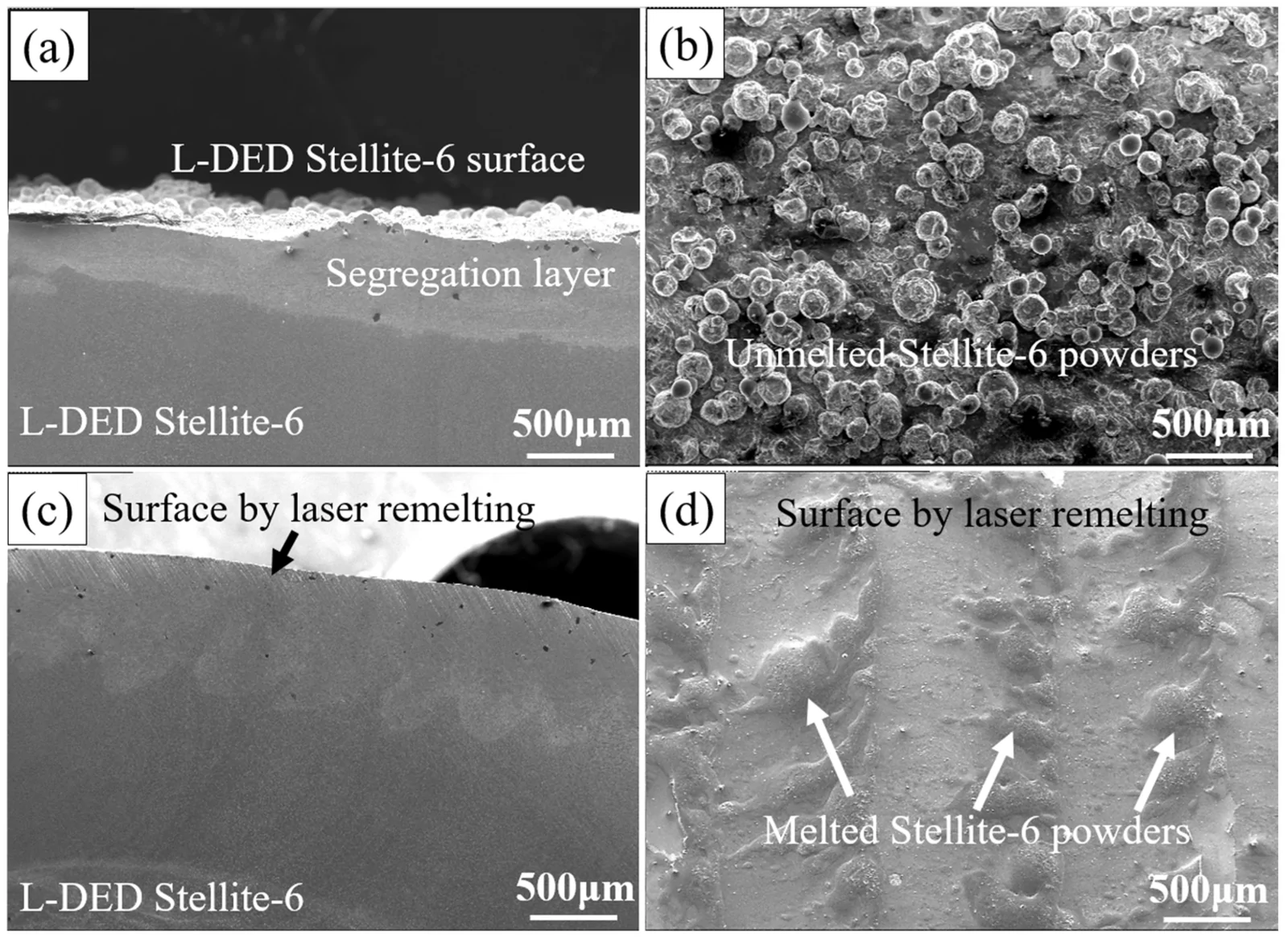
Cross-sectional and surface morphologies of the L-DED Stellite-6 surface layer before and after remelting: (**a**) Cross-sectional morphology of the non-remelted surface layer; (**b**) morphology of the non-remelted surface layer; (**c**) cross-sectional morphology of the remelted surface layer; (**d**) morphology of the remelted surface layer.

Figure 6a,b show the cross-sectional and surface morphologies of L-DED Stellite-6, respectively. As a large amount of unmelted powder remained on the specimen surface after L-DED, it was difficult to observe surface phase precipitation. Therefore, before thermal service, the surface was remelted using a low laser power of 300 W to melt the residual powder and improve surface flatness. Dye penetrant testing confirmed that no remelting-induced cracks were present. Figure 6c,d show the cross-sectional and surface morphologies after remelting. The near-surface segregation layer exhibits a serrated morphology, while its thickness remains close to 500 μm, nearly identical to that before remelting in Figure 3a. This indicates that low-power surface remelting did not significantly extend the depth range of short-range diffusion of Cr and C atoms in the surface layer of Stellite-6.

**Figure 7:**
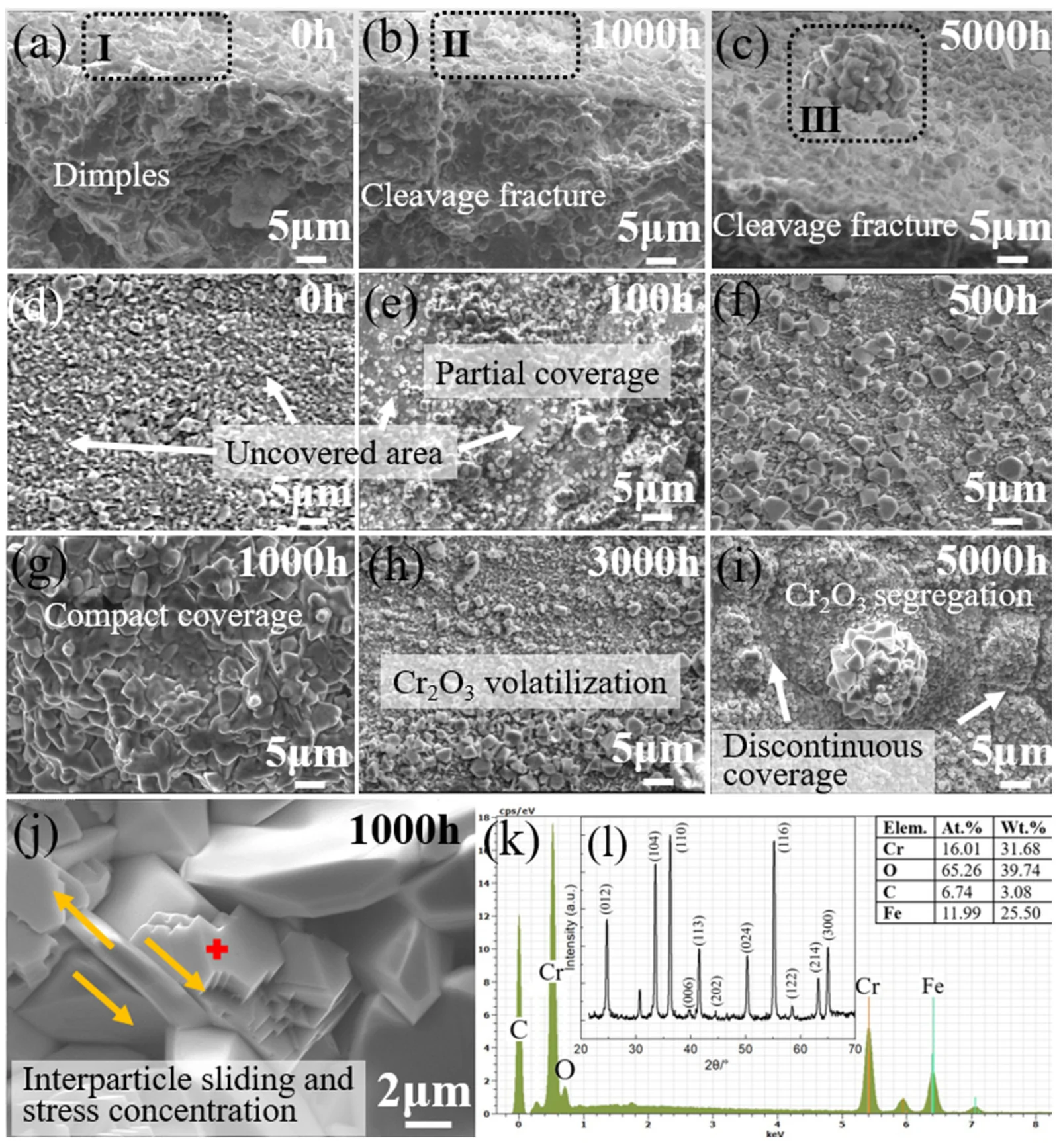
Microstructural morphologies of Cr_2_O_3_ in the surface layer and fracture surfaces during high-temperature service: (**a**) Fracture morphology after 100 h of service; (**b**) fracture morphology after 1000 h of service; (**c**) fracture morphology after 5000 h of service; (**d**–**i**) surface-layer morphologies after 0–5000 h of service; (**j**) growth morphology of spinel-structured Cr_2_O_3_; (**k**) EDS analysis of Cr_2_O_3_; (**l**) XRD analysis of Cr_2_O_3_.

Figure 7 shows the SEM and EDS results of the surface fracture and surface regions of L-DED Stellite-6 during service. Figure 7a–c show the evolution of the surface impact-fracture morphology from 0 to 5000 h. In as-built condition (0 h), numerous dimples are observed on the surface fracture, indicating ductile fracture. After 1000 h of service, pronounced transgranular fracture features appear on the surface fracture, and cleavage fracture remains dominant up to 5000 h.

Figure 7d–i show the morphological evolution of surface Cr_2_O_3_ from 0 to 5000 h. Figure 7d, a magnified view of region I in Figure 7a, shows that during 0–100 h of service, the surface is locally covered by a discontinuous Cr_2_O_3_ layer. At 500 h, as shown in Figure 7f, the surface region is extensively covered by Cr_2_O_3_ precipitates, and some Cr_2_O_3_ particles begin to grow markedly into spinel-like morphology. Figure 7g, a magnified view of region II in Figure 7b, shows the surface morphology after 1000 h of service, where the surface is densely covered by a Cr_2_O_3_ layer. Figure 7j shows the microscopic morphology of Cr_2_O_3_ particles after 1000 h, exhibiting a serrated, spinel-like, densely interlocked morphology with layered growth. The corresponding EDS result in Figure 7k indicates a Cr–O-dominated compound partially doped with Fe and C, which is typical of FeCr_2_O_4_ formed by the reaction between Cr_2_O_3_ and Fe oxides. Figure 7l presents the XRD analysis results of the Cr_2_O_3_ layer shown in Figure 7j after 1000 h of service. Figure 7h shows the surface morphology after 3000 h of service. Owing to the high-temperature volatilization of Cr_2_O_3_, the coating coverage density is significantly lower than that after 1000 h, resulting in the discontinuous surface layer observed after 5000 h in Figure 7i.

### 3.3. Impact Toughness and Subsurface Hardness Analysis

**Figure 8:**
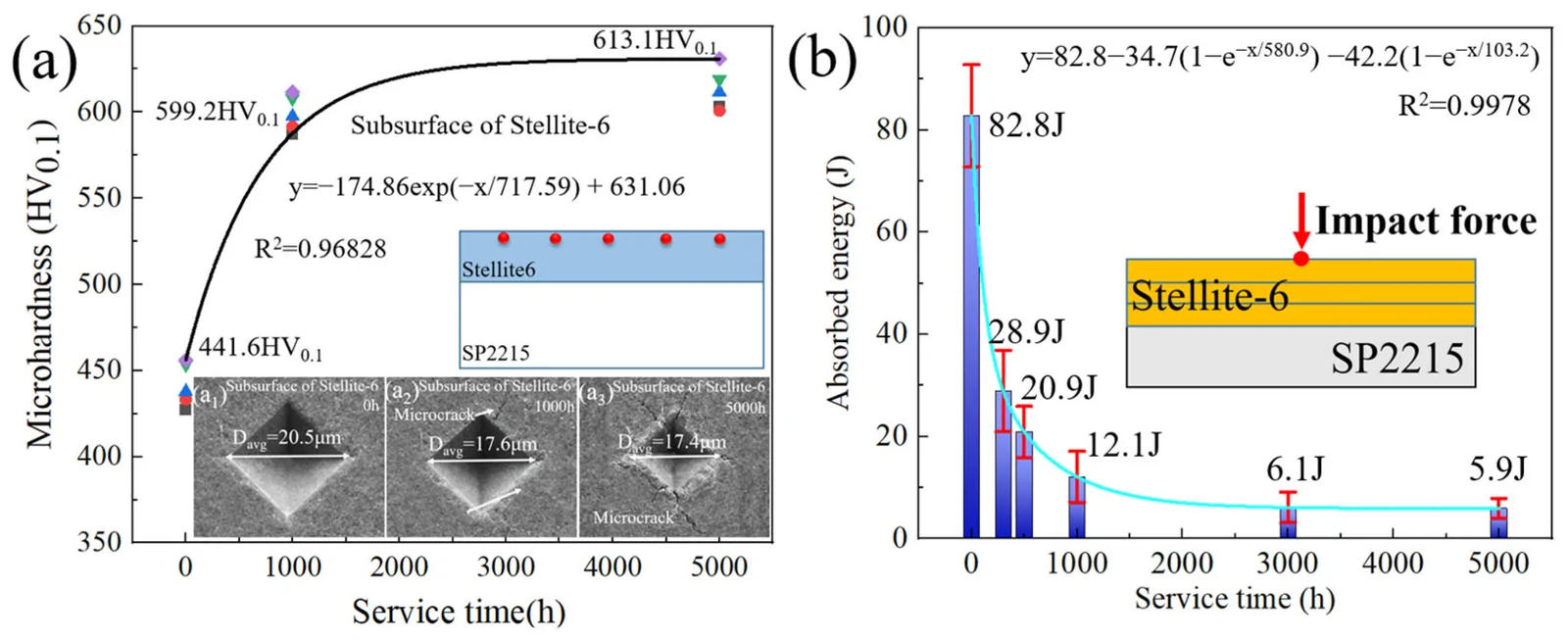
Evolution of subsurface hardness and overall impact-absorbed energy of L-DED SP2215/Stellite-6 during service: (**a**) hardness; (**a_1_**) 0 h; (**a_2_**) 1000 h; (**a_3_**) 5000 h (**b**) impact-absorbed energy.

Figure 8a shows the variation in microhardness of the L-DED Stellite-6 subsurface region with service time, together with the corresponding indentation morphologies. Five measurements were taken within each region, and their mean value was adopted for further analysis. In accordance with GB/T 4340.1–2024 and ISO 14577, microhardness testing was carried out at a peak load of 0.9807 N (HV_0.1_). The loading, dwell, and unloading stages were each maintained for 60 s to allow for adequate deformation and to enhance the reliability and accuracy of the hardness measurements.

Figure 8(a_1_–a_3_) show the hardness indentation morphologies after 0 h, 1000 h, and 5000 h of service, respectively. With increasing service time, the indentation diagonal lengths in the L-DED Stellite-6 subsurface region were 20.5 μm at 0 h, 17.6 μm at 1000 h, and 17.4 μm at 5000 h. The corresponding microhardness values were 441.6 HV_0.1_, 599.2 HV_0.1_, and 613.1 HV_0.1_, respectively. These results indicate that the subsurface hardness of L-DED Stellite-6 gradually increases with service time.

Figure 8b shows the unnotched impact-absorbed energy curves of the L-DED-fabricated SP2215/Stellite-6 samples after service for 0, 300, 500, 1000, 3000, and 5000 h. Three specimens were tested for each service duration. The impact-absorbed energies and corresponding fracture modes were 82.8 ± 4.2 J at 0 h, corresponding to ductile fracture; 28.9 ± 3.6 J at 300 h, corresponding to intergranular quasi-cleavage fracture; and 5.9 ± 2.5 J at 5000 h, corresponding to transgranular cleavage fracture. The overall decrease in absorbed energy reached 92.9%. These results demonstrate that the impact-absorbed energy of the component decreases progressively with increasing service time.

### 3.4. Surface Cracking Mechanism During Service

**Figure 9:**
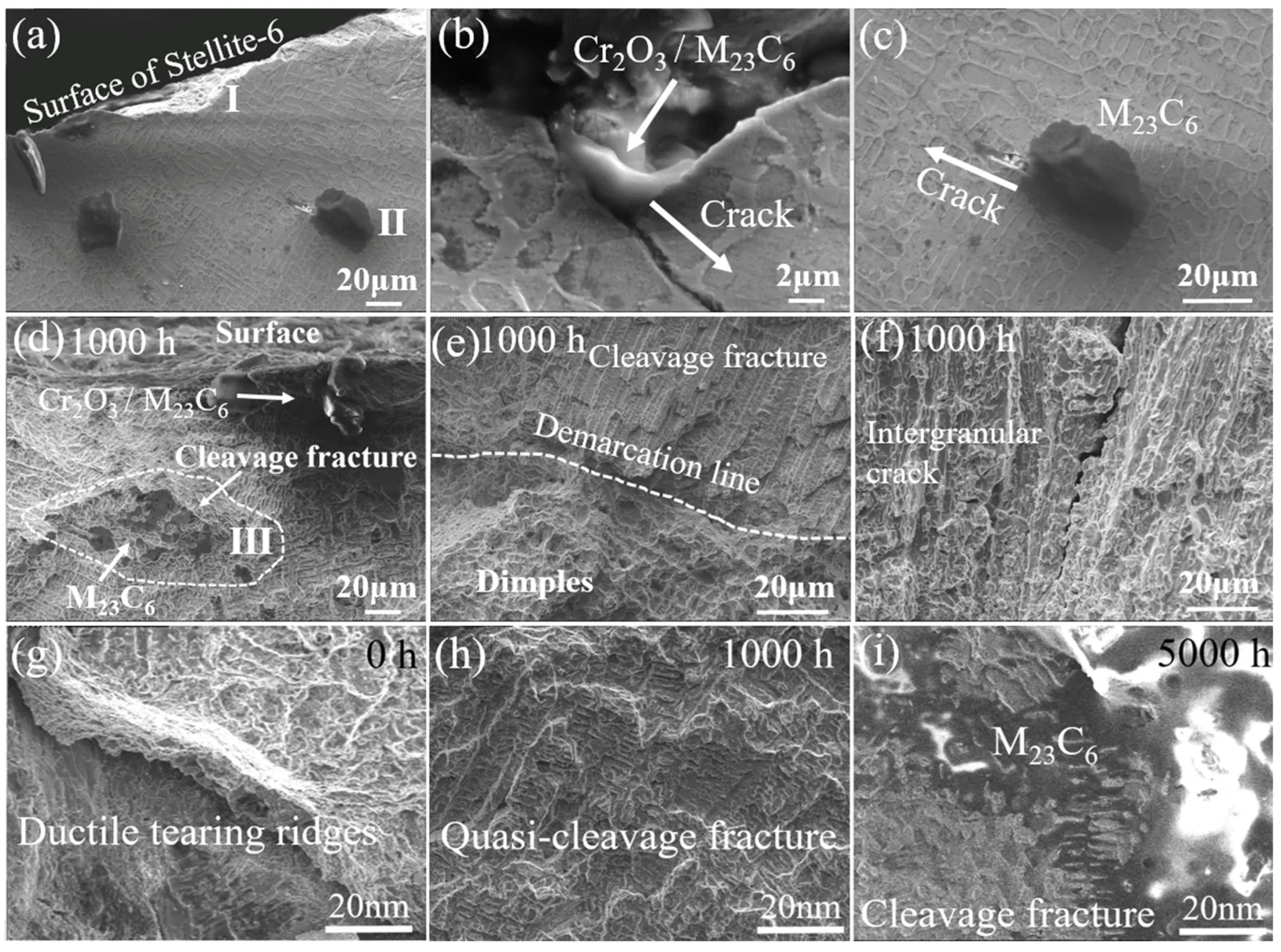
Surface cracking morphology after 1000 h of service: (**a**) Surface-layer morphology of L-DED Stellite-6; (**b**) M_23_C_6_/Cr_2_O_3_ precipitation morphology in the surface layer, corresponding to the magnified region I; (**c**) subsurface M_23_C_6_ precipitation morphology, corresponding to the magnified region II; (**d**) cleavage fracture morphology of the surface layer; (**e**) demarcation line between cleavage/ductile fracture; (**f**) mixed transgranular and intergranular cracks in the surface layer; (**g**–**i**) surface fracture morphologies after 0 h, 1000 h, and 5000 h of service.

Figure 9 shows the morphology near the fracture surface of the L-DED Stellite-6 impact specimen during service. Figure 9a–f,h depict the cracking morphologies in the surface region after 1000 h of service. Figure 9a presents the cross-sectional morphology of the surface layer, where a large number of coarse M_23_C_6_ precipitates larger than 20 μm are observed. Figure 9b shows a magnified view of region I. The coupled precipitation of M_23_C_6_/Cr_2_O_3_ phases in the surface layer induces stress concentration and promotes crack propagation into the coating. Figure 9c shows a magnified view of region II. In the subsurface region approximately 150 μm below the surface, coarse M_23_C_6_ phases reduce the wettability between particles and the matrix, thereby inducing local cracking.

Figure 9d–f show the impact-fracture morphologies of the surface and subsurface regions of L-DED Stellite-6. Figure 9d shows cleavage fracture features in both regions: the surface layer is enriched with coupled M_23_C_6_/Cr_2_O_3_ precipitates, whereas the subsurface region is dominated by M_23_C_6_ precipitation. Figure 9e, a magnified view of region III, shows pronounced cleavage fracture in the segregation region, while the lower region without obvious precipitation still retains partial dimple features characteristic of quasi-cleavage fracture. A clear cleavage/ductile fracture boundary is observed between the segregation and non-segregation regions. Figure 9f shows that, within the cleavage-fracture region, mixed transgranular and intergranular cracks form locally in the surface Stellite-6 layer after impact. Figure 9g–i show the surface fracture morphologies after 0 h, 1000 h, and 5000 h of service, respectively. With the progressive enrichment of M_23_C_6_ in the surface region, the fracture mode gradually changes from ductile fracture to cleavage fracture.

## 4. Discussion

On the basis of the above results, the evolution of the near-surface microstructure of SP2215/Stellite-6 components during prolonged service at 650 °C for up to 5000 h was systematically examined. Variations in surface hardness, impact-absorbed energy, and cracking behavior were also identified. The following section discusses these observations in terms of the associated microstructural evolution, thermo-mechanical coupling effects, and mechanisms governing surface failure.

### 4.1. Ripening Mechanism of M_23_c_6_ and Cr_2_o_3_ Phases in the Surface

**Figure 10:**
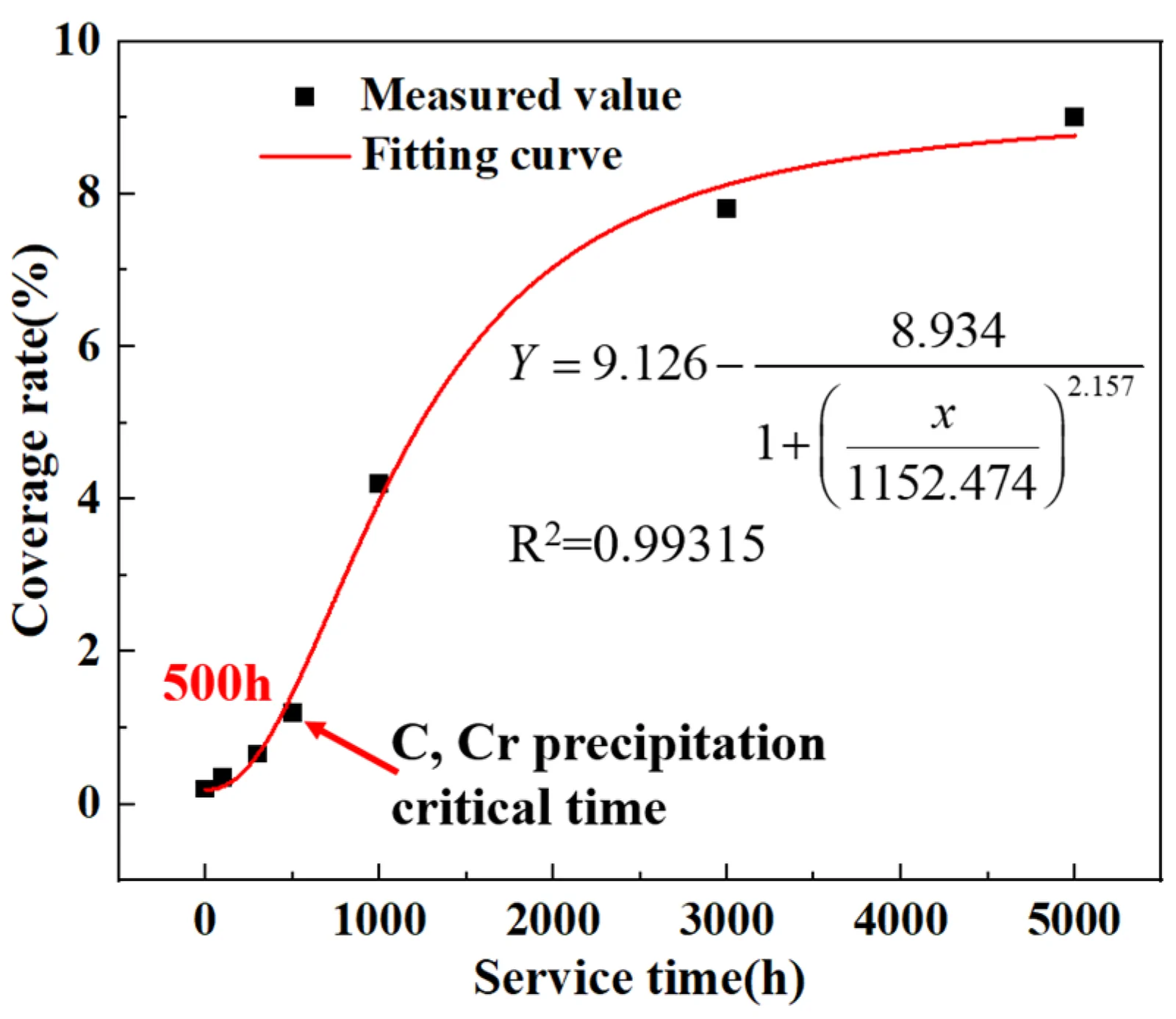
Variation in the coverage of the subsurface M_23_C_6_ phase with service time.

Figure 10 schematically illustrates the decomposition of Stellite-6 dendrite-boundary precipitates and atomic migration toward the surface during service. According to the analyses in Figure 2 and Figure 3, the M_23_C_6_ phase at the Stellite-6 dendrite boundaries undergoes unstable decomposition during service, increasing the concentration of mobile Cr, C, and W atoms within the dendrites and promoting their extensive migration from the bottom of the near-surface segregation layer toward the surface.

The atomic migration mechanism during L-DED solidification can be explained by vacancy formation and concentration-gradient effects. Owing to the rapid solidification of L-DED Stellite-6, vacancies do not have sufficient time to diffuse out. Vacancies that would normally migrate to grain boundaries or surfaces and disappear during slow cooling are instead frozen into the lattice. Meanwhile, rapid solidification shrinkage and post-solidification residual stress in the surface layer further promote vacancy clustering at the atomic scale. Once surface vacancies form, a diffusion driving force is generated between the highly mobile Cr and C atoms in the Stellite-6 layer and the surface vacancies, thereby promoting atomic migration toward the surface. During this process, because the interstitial diffusion rate of C (0.077 nm) is much higher than the substitutional diffusion rate of Cr (0.128 nm), C preferentially reaches the surface region of L-DED Stellite-6 and forms C-rich regions. As a result, the Cr, C, and W contents in the as-deposited Stellite-6 surface layer are significantly higher than those inside the coating.

In Figure 5, EDS analysis of the C-rich surface region showed that the atomic fractions of C, Cr, and W were approximately 53%, 28%, and 10%, respectively, with the remainder consisting mainly of Co and small amounts of other alloying elements. If Cr and W are considered the major metallic elements enriched in this region, the experimentally measured metal-to-carbon atomic ratio (M/C) is approximately 0.72, which is substantially lower than the theoretical stoichiometric ratio of 23/6 ≈ 3.83 for M_23_C_6_ and corresponds to only approximately 18.8% of the theoretical value. In other words, relative to the stoichiometric composition of M_23_C_6_, this region exhibits an approximately 81% relative deficiency in metallic atoms. Such a pronounced deviation from the stoichiometric ratio indicates that, during the early stage of service, C migrates substantially faster than Cr and W and preferentially accumulates in the surface region, whereas the migration of the metallic elements is significantly delayed.

This asynchronous elemental migration can be explained by the different diffusion mechanisms of C and Cr/W. As an interstitial solute atom, C migrates predominantly through interstitial diffusion and therefore has a relatively low diffusion activation energy. Previous studies have reported an activation energy of approximately 154 kJ/mol for the interstitial diffusion of C in relevant Co-based systems. In contrast, the diffusion of substitutional elements such as Cr is vacancy-mediated and has a substantially higher activation energy of approximately 255 kJ/mol. The difference in their activation barriers therefore exceeds 100 kJ/mol. Consequently, during prolonged high-temperature service at 650 °C, C retains a relatively high diffusion capability, whereas the substitutional diffusion of Cr and W is strongly restricted by the energetic requirements associated with vacancy formation and migration [30].

Based on the Arrhenius diffusion equation, the ratio between the diffusion coefficients of C and Cr can be expressed as [31]:

(1) $$\frac{D_{c}}{D_{\mathrm{Cr}}}=\exp(\frac{Q_{\mathrm{Cr}}-Q_{c}}{R\cdot T})$$

where T is the absolute temperature, equal to 923.15 K at 650 °C, and R is the universal gas constant, 8.314 J/(mol·K). The diffusion activation energies of interstitial C in the Co matrix and substitutional Cr in fcc-Co are approximately 154 and 255 kJ/mol, respectively. Based on the Arrhenius relationship and the corresponding diffusion parameters, their diffusion coefficients at 650 °C are estimated to be approximately 5.9 × 10^−14^ and 3.5 × 10^−20^, respectively. Thus, the diffusivity of C is approximately six orders of magnitude higher than that of Cr.

Therefore, even though C and metallic atoms are simultaneously released during the decomposition of precipitates such as M_23_C_6_, C can preferentially migrate toward and accumulate in defect-rich regions near the surface because of its substantially higher diffusivity. In contrast, the much slower substitutional diffusion of Cr and W prevents these elements from migrating synchronously with the rapidly diffusing C atoms. This pronounced difference in diffusion kinetics results in kinetic decoupling between C and Cr/W during elemental migration, ultimately producing a transient compositional characteristic of preferential C enrichment followed by delayed Cr/W migration.

This kinetic difference becomes particularly pronounced at the relatively low long-term service temperature of 650 °C. Owing to its lower diffusion activation barrier, C can retain appreciable interstitial mobility, whereas the migration of substitutional Cr and W is strongly constrained by their substantially higher diffusion barriers. This kinetic difference therefore provides a reasonable explanation for the experimental observations in the present study: during the early stage of service, C preferentially reaches and accumulates in the surface region, whereas the migration of Cr and W is significantly delayed. Cr and W subsequently migrate toward the pre-existing C-rich regions and participate in the formation and growth of Cr–C-rich precipitates.

During service, residual stress in the surface layer is relieved. Thus, the driving force for atomic migration toward the surface is no longer dominated by as-deposited surface vacancies but by the decomposition of inherent Stellite-6 dendrite-boundary precipitates and secondary-phase precipitation in the surface layer. As shown in Figure 3b,c, the formation of C-rich regions and C dispersion markedly accelerate the continuous decomposition of Stellite-6 dendrite-boundary precipitates near the surface, further increasing the local Cr and C contents and producing a clear morphological difference from the dendrite boundaries in the non M_23_C_6_-rich precipitation zone. In addition, thermal exposure of the L-DED Stellite-6 surface region during service promotes the reaction between Cr and O to form Cr_2_O_3_, which consumes surface Cr, lowers the local Cr concentration, and generates vacancies. The resulting concentration gradient drives continuous outward diffusion of Cr from the subsurface region to balance the gradient and replenish vacancies. Meanwhile, as indicated by the analysis in Figure 7, progressive volatilization of Cr_2_O_3_ during service continuously promotes Cr replenishment from the interior toward the surface. This Cr then combines with C accumulated in the subsurface region, inducing heterogeneous nucleation and growth of M_23_C_6_ carbides in the subsurface Stellite-6. With increasing aging time, the M_23_C_6_ precipitation zone continuously advances inward and thickens, corresponding to the continuous dark-contrast region observed in the surface layer in Figure 3.

Figure 10 shows the variation in the coverage of the subsurface M_23_C_6_ precipitation zone with service time and the corresponding fitting equation. The M_23_C_6_ coverage increases rapidly within 0–1000 h and then increases slowly during 3000–5000 h. The effect of service time on the surface M_23_C_6_ coverage approximately follows a gradually increasing Boltzmann-type trend.

**Figure 11:**
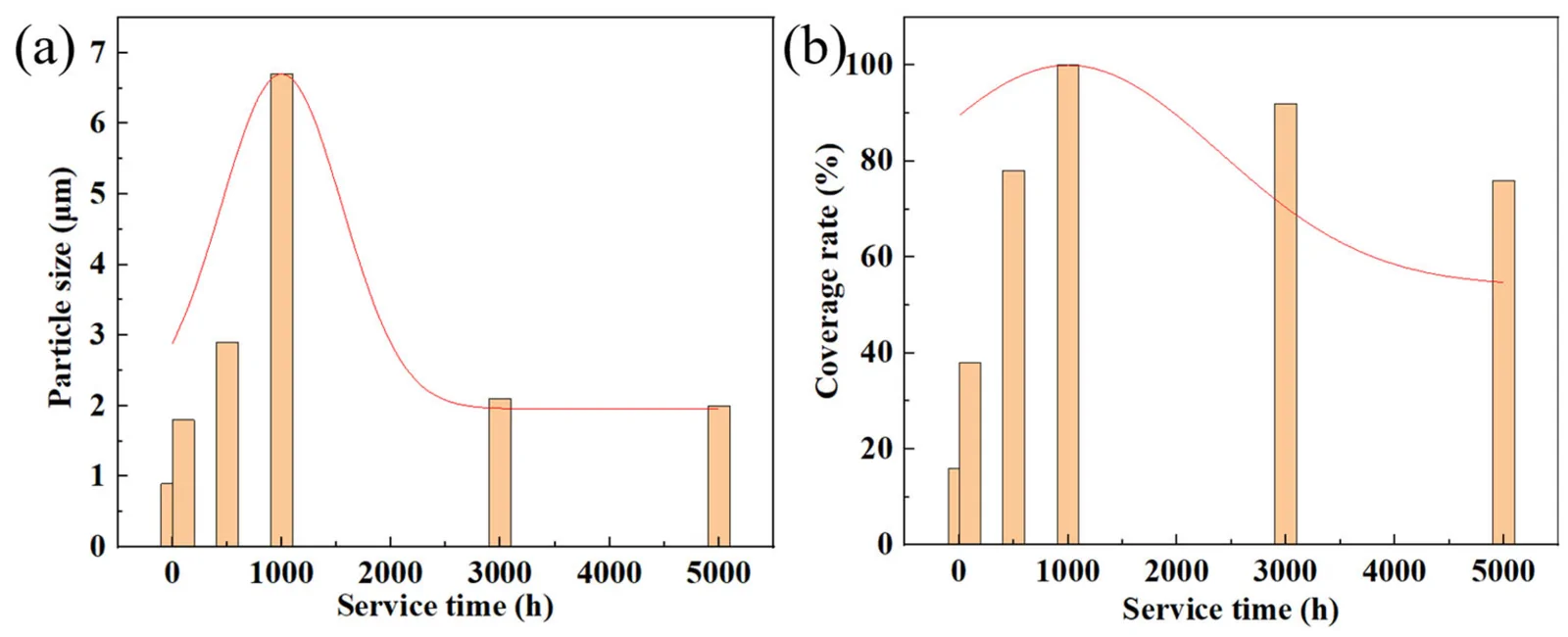
Evolution of surface Cr_2_O_3_ size and coverage in L-DED Stellite-6 during service: (**a**) particle size; (**b**) coverage rate.

Figure 11 shows the evolution curves of the size and coverage of surface Cr_2_O_3_ in L-DED Stellite-6 during service. Both the size and coverage of surface Cr_2_O_3_ vary with service time in an approximately normal-distribution trend, with the peak occurring at 1000 h. Compared with Figure 10, the difference between the coverage evolution curves of surface Cr_2_O_3_ and M_23_C_6_ can be attributed to selective oxidation. The affinity between Cr and O is much higher than that between Cr and C, causing a dense Cr_2_O_3_ layer to form preferentially on the surface. This layer improves high-temperature oxidation resistance, protects C in the subsurface region, and suppresses its outward escape, thereby leading to a gradual increase in the thickness of the surface M_23_C_6_ precipitation layer with increasing thermal service time.

Meanwhile, the high-temperature volatility of surface Cr_2_O_3_ promotes continuous Cr consumption in the Stellite-6 surface layer, reducing the local Cr concentration and generating atomic vacancies. The resulting concentration gradient drives continuous outward diffusion of Cr from the subsurface region to balance the concentration gradient and replenish vacancies, maintaining the surface Cr content in a dynamic equilibrium. During 0–1000 h of service, Cr_2_O_3_ precipitation on the surface is much stronger than volatilization, resulting in an overall increase in Cr_2_O_3_ coverage. After 1000 h, owing to the elemental barrier effect in the surface region, the reduced migration rate of Cr atoms lowers the surface Cr concentration. Consequently, Cr_2_O_3_ volatilization exceeds precipitation, leading to a decrease in Cr_2_O_3_ coverage.

The growth behavior of M_23_C_6_ and Cr_2_O_3_ in the surface layer of L-DED Stellite-6 during service, as shown in Figure 3 and Figure 7, can be explained by Ostwald ripening. Based on Figure 10 and Figure 11, the coverage growth rates of M_23_C_6_ and Cr_2_O_3_ in the Stellite-6 surface layer during the first 1000 h of service are calculated to be 0.004%·h^−1^ and 0.084%·h^−1^, respectively. During 5000 h of service, the corresponding rates decrease to 0.00176%·h^−1^ and 0.012%·h^−1^, respectively. These results indicate that the coverage of surface M_23_C_6_ and Cr_2_O_3_ increases rapidly within the first 1000 h and then gradually approaches a stable state during long-term service.

The rapid ripening of surface M_23_C_6_ and Cr_2_O_3_ is essentially driven by the high interfacial energy of fine precipitates caused by the curvature effect, namely the Gibbs–Thomson effect. To reduce interfacial energy and approach thermodynamic equilibrium, phase decomposition generates a driving force for atomic diffusion. Consequently, Cr and C atoms diffuse from the Stellite-6 matrix to the surfaces of larger particles in the surface layer, promoting their coarsening. According to the Lifshitz–Slyozov–Wagner (LSW) theory of phase coarsening [32], the variation in the average precipitate radius with service time can be predicted using the following growth kinetic equation [33]:

(2) $$r^{3}-r_{0}^{3}=\frac{8\gamma_{P}C_{e}V_{m}^{2}D}{9RT}t=Kt$$

Here, *t* denotes the service duration, *r* represents the mean precipitate radius at time *t*, and *r*_0_ is the initial particle radius before service. *γ* is the interfacial energy between the coarsened precipitates and the matrix, *C*_e_ denotes the equilibrium solute concentration corresponding to an infinitely large particle, *R* is the Boltzmann constant, *T* is the service temperature, *V*_m_ is the molar volume of the precipitate, *K* represents the LSW coarsening-rate constant, and *D* denotes the diffusion coefficient governing solute transport.

Accordingly, precipitate coarsening is primarily driven by the tendency to reduce the interfacial free energy between the particles and the surrounding matrix. With increasing service duration, the mean radius r of the M_23_C_6_ precipitates progressively increases, and according to LSW theory, *r*^3^ varies linearly with service time *t*.

### 4.2. Failure Mechanisms of L-Ded Stellite-6 Surface

According to the analysis in Section 4.1, the coverages of Cr_2_O_3_ in the surface layer and M_23_C_6_ in the subsurface region of L-DED Stellite-6 gradually increase during long-term high-temperature service. With the increase in precipitate size, the dispersion-strengthening effect gradually weakens, whereas structural strengthening induced by coarse M_23_C_6_ and Cr_2_O_3_ becomes increasingly dominant. Combined analysis of Figure 8a and Figure 10 indicates that this process significantly increases the surface hardness, showing a positive correlation between them.

The coverage and size of surface precipitates are governed by the nucleation rate and growth rate, respectively. Based on the decomposition of M_23_C_6_ at Stellite-6 grain boundaries during service, the number of nuclei formed by M_23_C_6_ and Cr_2_O_3_ phases in the near-surface region per unit time and unit volume can be expressed as follows [34]:

(3) $$N=K_{1}\exp\left(\frac{-K_{2}}{kT{\left(\Delta G^{*}\right)}^{2}}\right)\cdot \exp\left(\frac{-Q}{kT}\right)$$

where *K*_1_ is the nucleation proportionality constant, *K*_2_ is the nucleation interface-related constant, ΔG* is the critical nucleation driving force, *Q* is the activation energy for diffusion of Cr atoms at the L-DED Stellite-6 interface, *K* is the Boltzmann constant, and *T* is the thermodynamic temperature.

Based on the analyses in Figure 3 and Figure 7, the high concentrations of Cr and C atoms in the Stellite-6 layer during service significantly reduce the diffusion activation energy *Q*, thereby increasing the nucleation rate *N* of surface M_23_C_6_ and Cr_2_O_3_. This consequently enlarges the distribution area of M_23_C_6_ and Cr_2_O_3_ phases in the surface layer. Meanwhile, the growth rate of the precipitates can be expressed as follows [35]:

(4) $$v=\frac{D}{\left|C_{\beta }-C_{\alpha }\right|}\left(\frac{\partial C}{\partial x}\right)$$

where *v* is the growth rate of the M_23_C_6_ phase, *C_α_* and *C_β_* are the Cr concentrations in the matrix and the new phase, respectively, *D* is the diffusion coefficient of Cr atoms, and ∂C/∂x is the change in Cr concentration within the interface-advance distance per unit area and unit time.

Based on the analysis in Section 4.1, the high concentrations of Cr and C atoms during service increase the diffusion coefficient *D* of Cr, thereby accelerating its migration toward the surface. Meanwhile, according to the Gibbs–Thomson effect, the high concentrations of Cr and C atoms, together with O atoms supplied from the surface atmosphere, markedly increase the growth rate *v* of M_23_C_6_ and Cr_2_O_3_ in the surface layer.

Therefore, according to Equations (3) and (4), high-temperature service significantly increases both the nucleation rate *N* and growth rate *v* of M_23_C_6_ in the surface region of L-DED Stellite-6. Based on the Johnson–Mehl kinetic equation, assuming spherical M_23_C_6_ particles with constant nucleation and growth rates, the volume fraction transformed at a given temperature of 650 °C can be expressed as follows [36]:

(5) $$\omega (t)=1-\exp(-\frac{\pi }{3}Nv^{3}t^{4})$$

where *N* is the nucleation rate, and *v* is the growth rate.

Clearly, longer service time leads to higher values of *N* and *v*, thereby increasing the volume fraction transformed for M_23_C_6_. However, for the non-spherical growth of Cr_2_O_3_, this transformation behavior can be described using the generalized Avrami equation derived from the Johnson–Mehl–Avrami–Kolmogorov (JMAK) model [37]:

(6) $$\varphi =1-\exp(-qt^{n})$$

where *q = f (N, v)* is a composite constant related to the nucleation rate *N* and growth rate *v*. In the classical Johnson–Mehl model with a constant nucleation rate, *q ∝ N·v*^3^. This equation is no longer limited to a specific precipitate morphology; instead, the Avrami exponent *n* comprehensively reflects the nucleation mechanism and growth mode. Therefore, higher *N* and *v* also promote an increase in the transformed fraction of spinel-structured Cr_2_O_3_ on the L-DED Stellite-6 surface.

**Figure 12:**
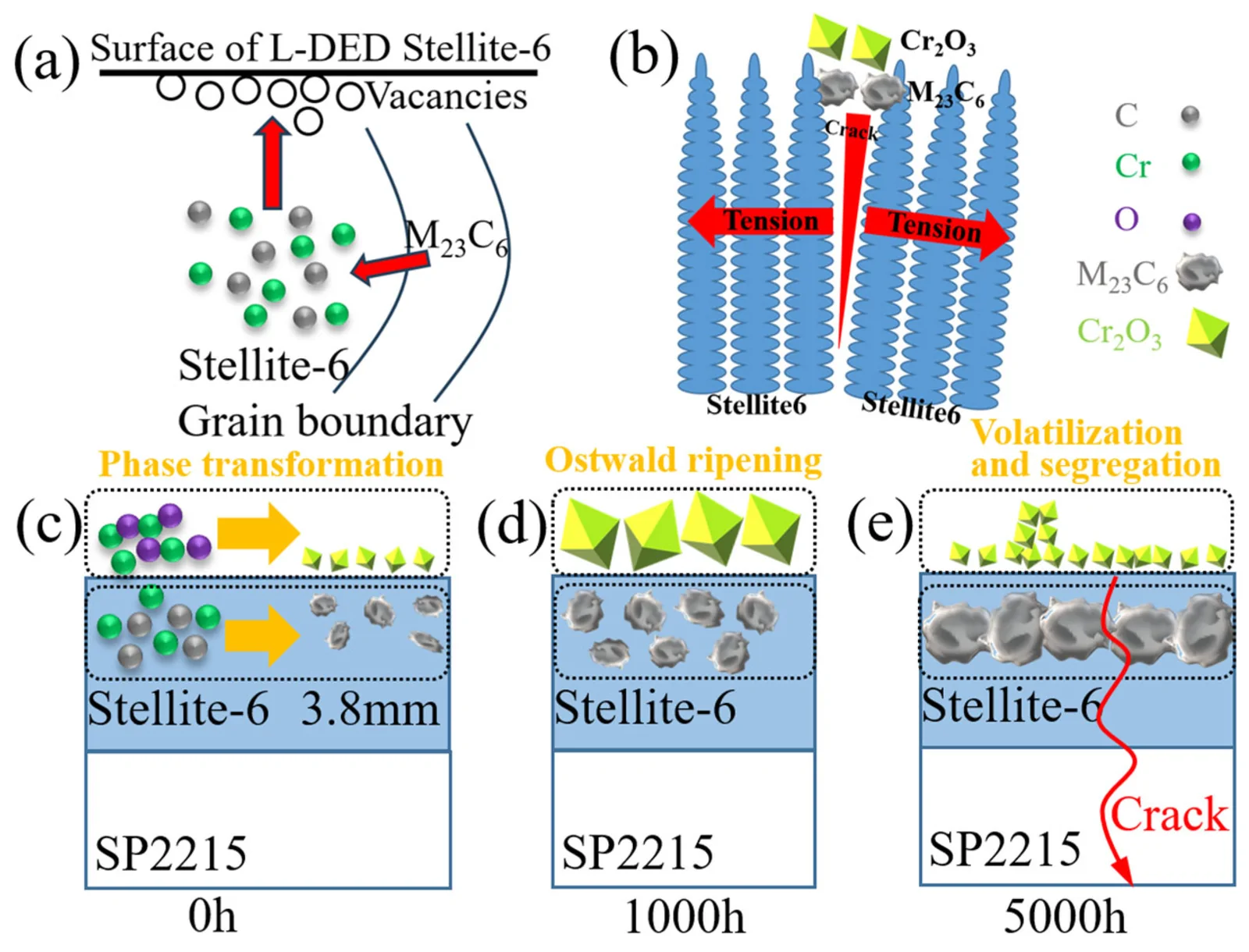
Schematic illustration of precipitation and cracking on the surface of L-DED Stellite-6 during service: (**a**) Decomposition of dendrite-boundary precipitates and atomic migration; (**b**) cracking mechanism induced by M_23_C_6_/Cr_2_O_3_; (**c**–**e**) precipitation mechanism of surface M_23_C_6_ and Cr_2_O_3_.

Figure 12b–e schematically illustrate the ripening of Cr_2_O_3_ and M_23_C_6_ on the L-DED Stellite-6 surface during service and the resulting surface crack initiation.

As shown in Figure 7j, the spinel-structured Cr_2_O_3_ in the surface layer exhibits face-to-face bonding between particles. Under stress loading in the Stellite-6 surface layer, interfacial misalignment and relative sliding occur between these particles. Moreover, the spinel structure induces pronounced stress concentration in the surface microstructure, leading to microcrack initiation in the surface layer of L-DED SP2215/Stellite-6 tube-wall components during service and subsequent inward propagation.

When the microcracks reach the M_23_C_6_ precipitation zone in the subsurface layer of L-DED Stellite-6, crack propagation changes into opening-mode growth as the interphase spacing of chain-like M_23_C_6_ gradually increases [38,39]. An approximate analysis can be performed using the Irwin model within the framework of linear elastic fracture mechanics (LEFM), assuming small-scale yielding at the crack tip. According to Irwin’s crack-tip plastic-zone model, under opening-mode, namely mode I, loading, the core relationship between the crack-tip opening displacement *δ* and the stress intensity factor *K_I_* can be expressed as follows [40]:

(7) $$\delta \approx K_{I}^{2}/(E\cdot \sigma_{y})$$

where *E* is the elastic modulus and *σ_y_* is the yield strength.

Meanwhile, according to the LEFM definition of the stress intensity factor, *K_I_* = *Yσ_f_* (*πa*)^1/2^. The fracture criterion is that unstable crack propagation and fracture occur when *K_I_* reaches the fracture toughness *K_IC_* of the material. Therefore, the relationship between the crack-tip opening displacement *δ* and the effective crack size *a* can be expressed as follows [40]:

(8) $$\delta \propto \sigma_{f}^{2}a/(E\cdot \sigma_{y})$$

where *σ_f_* is the applied stress and *Y* is the geometry factor related to the component and crack configuration.

According to the analyses based on Equations (2)–(5), together with the results in Figure 3 and Figure 12, the interphase void size caused by M_23_C_6_ coarsening, namely the effective crack size *a*, continuously increases with service time. Under the same applied stress *σ_f_*, an increase in *a* leads to a larger *δ*, indicating that the subsurface M_23_C_6_ precipitation zone facilitates crack propagation. Conversely, to reach the critical crack-opening displacement *δ_c_*, the required fracture stress *σ_f_* inevitably decreases as *a* increases.

Based on the analyses in Figure 8 and Figure 10, the thickness of subsurface M_23_C_6_ is negatively correlated with the toughness of L-DED SP2215/Stellite-6. This indicates that interphase voids associated with M_23_C_6_ during service not only act as crack sources and reduce the fracture strength (*σ_f_*) of the material but also intensify stress concentration under impact loading, promoting rapid crack propagation. This markedly reduces the plastic deformation energy consumed during crack initiation and growth, ultimately leading to a substantial decrease in impact-absorbed energy. These results demonstrate that increased interphase void size significantly deteriorates both the static fracture strength and dynamic impact toughness of the material. According to the results in Figure 8, each increase of 1 HV_0.1_ in surface hardness during service is accompanied by a decrease of 0.448 J in the impact-absorbed energy of the component, indicating a negative correlation between them.

This study establishes the relationship between M_23_C_6_ decomposition and the coarsening mechanisms of surface Cr_2_O_3_ and M_23_C_6_ during the high-temperature service of L-DED Stellite-6 coatings. Furthermore, it reveals the thermomechanical coupling between microstructural evolution and the degradation of surface hardness, fracture strength, and impact toughness in L-DED SP2215/Stellite-6 components. The results demonstrate that extensive Cr and C atomic migration and segregation induced by dendritic boundary M_23_C_6_ decomposition are the primary causes of surface embrittlement and cracking during service. These findings provide a theoretical basis for current efforts to optimize L-DED Stellite-6 composition, suppress service-induced elemental segregation and coarse M_23_C_6_ precipitation, and consequently enhance coating toughness. Moreover, the thermomechanical coupling mechanisms revealed in this study provide fundamental guidance for future investigations of other austenitic stainless steel/Co-based alloy interfaces, facilitating their broader industrial applications.

## 5. Conclusions

The microstructural evolution, surface hardness, and impact toughness of the near-surface region of L-DED SP2215/Stellite-6 were systematically investigated during isothermal service at 650 °C for up to 5000 h. The results establish a clear relationship among elemental redistribution, carbide/oxide evolution, surface hardening, embrittlement, and crack propagation. The main scientific and engineering conclusions are summarized as follows:

(1) During high-temperature service, the unstable M_23_C_6_ precipitates along the dendritic boundaries of Stellite-6 progressively decompose and release Cr and C atoms. The resulting concentration gradients drive these solute atoms toward the near-surface region, providing the fundamental source for subsequent surface and subsurface phase evolution.
(2) Owing to its higher interstitial diffusivity, C preferentially migrates to and accumulates in the subsurface region, while Cr/W migration is delayed. This promotes the formation of a C-rich zone and subsequent M_23_C_6_ enrichment, whereas Cr reaching the surface forms a Cr_2_O_3_-rich layer. The enrichment of subsurface M_23_C_6_ and surface Cr_2_O_3_ is the main microstructural origin of the increased surface hardness during service.
(3) The surface Cr_2_O_3_ layer mainly promotes crack initiation through local stress concentration and interfacial sliding, while the chain-like subsurface M_23_C_6_ precipitates govern subsequent crack propagation. Their coarsening and associated interparticle gaps provide preferential paths for microcrack growth into Mode I opening cracks. Therefore, M_23_C_6_-induced embrittlement is the primary cause of the pronounced loss of impact toughness during long-term service.
(4) From an engineering perspective, suppressing Cr/C redistribution, M_23_C_6_ coarsening, and surface Cr_2_O_3_ accumulation is essential for improving the long-term cracking resistance of L-DED Stellite-6 coatings. However, the present conclusions are limited to the investigated SP2215/Stellite-6 configuration at 650 °C. Future work should evaluate compositional/gradient design and functional interlayers while further verifying reproducibility and scalability across different deposition batches, coating thicknesses, and component scales.
(5) The present thermal-service condition reproduces long-term exposure at 650 °C but does not fully represent the coupled thermo-mechanical-corrosive environment of actual boiler operation. Future work will include in-boiler exposure, oxidation, erosion, thermal cycling, and through-wall thermal stresses to further validate the long-term applicability of the proposed mechanism and improve service-life assessment under realistic conditions.

## Figures and Tables

**Table 1:** Chemical composition of SP 2215 stainless steel and Stellite 6 powder (wt.%).

Elem.	C	Si	Mn	Cr	Ni	Cu	Nb	Mo	W	N	Al	Fe	Co
SP 2215	0.072	0.38	0.62	22.46	15.44	3.44	0.49	0.12	0.03	0.26	0.02	Bal.	0.06
Stellite 6	1.28	1.49	0.15	29.33	2.27	—	—	0.85	5.10	—	—	2.31	Bal.

## Data Availability

The original contributions presented in this study are included in the article. Further inquiries can be directed to the corresponding author.
